# Selective Inhibition of Vascular Smooth Muscle Cell Function by COVID-19 Antiviral Drugs: Impact of Heme Oxygenase-1

**DOI:** 10.3390/antiox14080945

**Published:** 2025-07-31

**Authors:** Kelly J. Peyton, Giovanna L. Durante, William Durante

**Affiliations:** Department of Medical Pharmacology and Physiology, University of Missouri, Columbia, MO 65212, USA

**Keywords:** remdesivir, molnupiravir, nirmatrelvir, COVID-19, vascular smooth muscle, proliferation, migration, heme oxygenase-1

## Abstract

Coronavirus disease 2019 (COVID-19) causes cardiovascular complications, which contributes to the high mortality rate of the disease. Emerging evidence indicates that aberrant vascular smooth muscle cell (SMC) function is a key driver of vascular disease in COVID-19. While antivirals alleviate the symptoms of COVID-19, it is not known whether these drugs directly affect SMCs. Accordingly, the present study investigated the ability of three approved COVID-19 antiviral drugs to influence SMC function. Treatment of SMCs with remdesivir (RDV), but not molnupiravir or nirmatrelvir, inhibited cell proliferation, DNA synthesis, and migration without affecting cell viability. RDV also stimulated an increase in heme oxygenase-1 (HO-1) expression that was not observed with molnupiravir or nirmatrelvir. The induction of HO-1 by RDV was abolished by mutating the antioxidant responsive element of the promoter, overexpressing dominant-negative NF-E2-related factor-2 (Nrf2), or treating cells with an antioxidant. Finally, silencing HO-1 partly rescued the proliferative and migratory response of RDV-treated SMCs, and this was reversed by carbon monoxide and bilirubin. In conclusion, the induction of HO-1 via the oxidant-sensitive Nrf2 signaling pathway contributes to the antiproliferative and antimigratory actions of RDV by generating carbon monoxide and bilirubin. These pleiotropic actions of RDV may prevent occlusive vascular disease in COVID-19.

## 1. Introduction

Coronavirus disease 2019 (COVID-19) is caused by the severe acute respiratory syndrome coronavirus 2 (SARS-CoV-2). The infectious disease was first reported in Wuhan, China in December 2019 and spread rapidly globally, resulting in the deaths of over 7 million people [1]. While most cases of infection are asymptomatic or give rise to mild flu-like symptoms, a significant number of infections elicit an aberrant immune response that unleashes a cytokine storm leading to severe pulmonary inflammation and lung damage [2,3,4]. Moreover, the release of inflammatory cytokines into the circulation induces vascular cell dysfunction and increases in vascular tone, thrombosis, and fibrosis, which contributes to multiorgan failure and the high rates of mortality in COVID-19. Indeed, cardiovascular complications arise in approximately one in eight patients hospitalized with COVID-19 [5], while about 30% of all COVID-19 related deaths were as a result of cardiovascular disease [6,7].

While endothelial cell dysfunction has been implicated in the development of vascular disease in COVID-19 [8,9,10], emerging studies suggest that vascular smooth muscle cells (SMCs) also contribute to the vasculopathy observed in COVID-19. SMCs express high levels of angiotensin-converting enzyme 2, providing a route for the infection of SMCs by SARS-CoV-2 [11,12]. Moreover, treatment of SMCs with the recombinant SARS-CoV-2 spike protein S1 causes activation of growth signaling pathways, which may mediate the thickening of the vessel wall that is noted in COVID-19 patients [13,14]. SMCs from COVID-19 patients also exhibit increased expression of chondroitin sulfates, which contributes to arterial stiffness [15,16]. More recently, vascular SMCs from post-COVID-19 patients displayed hypercontractile responses and were less responsive to nitric oxide-induced relaxation [17]. Furthermore, histopathological findings and whole transcriptome studies implicate vascular SMC dysfunction and fibrosis as key mechanisms underlying cardiovascular complications in COVID-19 patients [17].

Although vaccination programs have dramatically reduced SARS-CoV-2 infection and disease severity, vaccination efficiency is compromised by the willingness of the population to get vaccinated and by the emergence of mutant viruses that initiates new outbreaks of infection, illustrating the need for additional therapeutic options in mitigating this deadly pandemic. In this respect, the recent development of novel antiviral drugs has impeded the viral spread within the patient and alleviated the symptoms of COVID-19. To date, the United States Food and Drug administration has approved the use of three antiviral drugs for the treatment of COVID-19 [18]. Remdesivir (RDV) was the first antiviral drug authorized to treat COVID-19. It is a phosphoramidate substituted nucleotide analog that acts as a viral RNA synthesis inhibitor by interfering with viral RNA-dependent RNA polymerase [19]. Molnupiravir (MPV) is another ribonucleoside prodrug that targets viral RNA-dependent RNA polymerase serving as a mutagenic agent rather than chain terminator leading to the accumulation of deleterious errors throughout the viral genome, rendering the virus noninfectious and non-replicative [20]. Finally, nirmatrelvir (NTV) is the active antiviral agent in paxlovid^TM^ that is also approved for use in COVID-19. NTV is a protease inhibitor that is active against 3C-like protease, the principal protease of SARS-CoV-2 that is essential for viral replication and blocks the necessary cleavage of the two viral polyproteins [21]. While these antiviral agents have been shown to be effective in reducing pulmonary viral titers and tissue pathology, potential off-target effects of these drugs remain largely unexplored. Moreover, the biological actions of these drugs on vascular cells have not been investigated.

Heme oxygenase-1 (HO-1) is an extremely inducible enzyme that metabolizes heme to carbon monoxide (CO), ferrous iron, and biliverdin, which is rapidly reduced to bilirubin by biliverdin reductase [22,23,24,25]. HO-1 plays an important role in preserving vascular health by diminishing oxidative stress, inflammation, and cell death in arteries. In addition, HO-1 exerts an antithrombotic effect by blocking platelet aggregation and adhesion [22]. Furthermore, HO-1 arrests vascular SMC proliferation and migration and mitigates intimal thickening and atherosclerosis by liberating CO and/or bilirubin [23,24,25]. Aside from its beneficial effects in circulation, HO-1 possesses potent antiviral effects against a host of viruses and has recently been proposed as a potential therapeutic target for COVID-19 [26,27,28,29]. Interestingly, previous studies from our laboratory and others found that human immunodeficiency virus protease inhibitors stimulate the expression of HO-1, raising the possibility that antiviral drugs may also mitigate the cardiovascular deficits associated with viral infections [30,31,32,33]. However, it is currently not known whether antiviral drugs that directly target SARS-CoV-2 regulate the expression of HO-1.

Given the putative role that vascular SMCs play in furthering vascular disease in COVID-19, the current study explored the effect of three distinct antiviral drugs directed against SARS-CoV-2 (RDV, MPV, and NTV) on vascular SMC growth and migration. Furthermore, as HO-1 directly modulates SMC function, the capacity of these antiviral agents to stimulate HO-1 expression was examined. Lastly, the role of HO-1 in mediating the cellular effects of these drugs was also ascertained.

## 2. Materials and Methods

### 2.1. Materials

Collagenase, elastase, minimum essential medium, penicillin, streptomycin, ethylenediaminetetraacetic acid (EDTA), trypsin, bovine calf serum, sodium dodecyl sulfate (SDS), trichloroacetic acid, phosphate buffered serum, NaOH, NADPH, MgCl_2,_ phosphate-buffered saline (PBS), acetic acid, glutamine, chloroform, CO-releasing molecule 2 (CORM2), hemin, glucose-6-phosphate, bromophenol blue, glucose-6-phosphate dehydrogenase, RNase, mercaptoethanol, formaldehyde, N-acetyl-L-cysteine were from Sigma Aldrich. Lipofectamine, Trizol, and 5-(and 6)-chloromethyl-2-7-dichlorodihydrofluorescein diacetate acetyl ester (CM-H_2_DCFDA) were from Life Technologies (Carlsbad, CA, USA). Polyclonal antibodies against HO-1 and HO-2 were from Enzo Life Sciences (Farmingdale, NY, USA) while antibodies directed against NF-E2-related factor-2 (Nrf2) and β-actin were from Santa Cruz Biotechnology (Santa Cruz, CA, USA). A polyclonal KLF2 antibody was from ThermoFischer Scientific (Waltham, MA, USA). Inactivated CORM2 (iCORM2) was made by leaving CORM2 solutions at room temperature for two days and then removing any remaining CO with a stream of nitrogen gas. [^3^H]Thymidine (20 Ci/mmol) was from Perkin Elmer (Boston, MA, USA). RDV was purchased from Cayman Chemical (Ann Arbor, MI, USA) and MPV, NTV, and NS-398 were from Selleckchem (Houston, TX, USA).

### 2.2. Cell Culture

Rat aortic SMCs were isolated, propagated, and characterized as we previously described [34]. Cells were maintained in minimum essential medium containing 10% bovine calf serum, Earle’s salts, 2 mM L-glutamine, 100 U/mL of penicillin, and 100 U/mL streptomycin. Human aortic SMCs were obtained from Cell Biologics (Chicago, IL, USA) and serially cultured in basal medium with insulin, epidermal growth factor, fibroblast growth factor, penicillin, streptomycin, amphotericin B, and hydrocortisone with 10% fetal bovine serum. Cells were propagated in a humidified atmosphere of 95% air and 5% CO_2_ at 37 °C. For the proliferation and migration experiments, cells were quiesced by putting rat aortic SMCs on serum-free medium containing bovine serum albumin (1%) and human aortic SMCs on basal medium without growth supplements for 48 h prior to treatment.

### 2.3. Cell Proliferation and DNA Synthesis

Cells were plated (2 × 10^4^ cells/well) onto 12-well plates in serum-containing media. Following quiescence, cells were treated with serum-complete media in the absence and presence of antiviral agents. During the treatment period, the media were replaced every 48 h with fresh serum-replete media containing the indicated concentration of antiviral drugs. After three days, cells were dissociating with trypsin (0.5%):EDTA (53 mM) and counted using an automated cell counter (Moxi Z ORFLO Technologies, Ketchum, ID, USA). In addition, DNA synthesis was monitored by pulsing SMCs with [^3^H]thymidine (1 µCi/mL) for four h. Cells were then washed with ice-cold PBS, fixed with 10% trichloroacetic acid, DNA extracted with 0.2% SDS/0.2 N NaOH, and radioactivity measured by scintillation counting (Tricarb liquid scintillation analyzer, model 2100, Packard, Meriden, CT, USA), as previously described [35].

### 2.4. Cell Migration

Cell migration was assessed via the scratch-wound assay [36]. Once a confluent cell monolayer was achieved, a sterile pipet tip was gently dragged across the cell layer generating a straight and uniform wound approximately 0.1 cm in width. Detached cells and debris were removed by several washes with PBS. Injured monolayers were then treated with hydroxyurea (2 mM) to block cell growth and exposed to various antiviral agents. Images of the damaged area were taken immediately and 24 h after injury with an inverted microscope fitted with a camera (Q-Imaging, QICAM; Hitschfel Instruments Incorporated, St. Louis, MO, USA). The degree of wound repair was monitored by planimetry.

### 2.5. Cell Viability

Cell viability was analyzed by measuring lactate dehydrogenase activity in the culture media using the CytoTox 96 Non-Radioactive Cytotoxicity Assay following the manufacturer’s protocol (Promega Life Sciences, Madison, WI, USA). In brief, culture media overlaying cells were collected and centrifuged at 3000× *g* for 10 min at room temperature. An aliquot of the supernatant was transferred to a 96-well plate containing an equal amount of CytoTox96 reagent. Following 30 min of incubation at room temperature, the reaction was stopped by adding acetic acid (1 M) and absorbance at 490 nm determined using a µQuant spectrophotometer (Bio-Tek Instruments, Winooski, VT, USA). Lactate dehydrogenase activity was stated as a percentage of control cells.

### 2.6. Western Blotting

Cells were washed, collected in electrophoresis buffer (125 mM Tris, pH 6.8, 12.5% glycerol, 2% SDS, and trace bromophenol blue), and proteins (20 µg) separated by SDS-polyacrylamide gel electrophoresis. Following electrophoretic transfer to nitrocellulose, membranes were blocked with 5% non-fat dried milk in PBS and then probed overnight at 4 °C with primary antibodies against HO-1 (1:1500), KLF2 (1:400), or β-actin (1:2000). Membranes were then washed four times with PBS and incubated with appropriate horseradish peroxidase-labeled secondary antibodies directed against mouse or rabbit IgG for 60 min at room temperature. Membranes were washed, immunoreactive bands visualized by enhanced chemoluminescence (GE Healthcare, Chicago, IL, USA), and captured by the Chemidoc^TM^ MP Imaging System (Bio-Rad Laboratories, Hercules, CA, USA). Image density was quantified using SigmaScan Pro 5 software (Systat Incorporated, Richmond, CA, USA), and normalized with respect to β-actin [37].

### 2.7. Quantitative Real-Time PCR (qRT-PCR) Analysis

Total RNA was extracted from cells with Trizol reagent (Thermo Fisher Scientific, Waltham, MA, USA). RNA content was determined spectrophotometrically at 260 nm and reversed transcribed to cDNA using the High-Capacity RNA-to-cDNA kit and random hexamer primers (Thermo Fisher Scientific, Waltham, MA, USA). qRT-PCR was executed with the SYBER Green Cycler iQ 5RT-PCR detection system (Bio-Rad Laboratories, Hercules, CA, USA). The primer sequences were: HO-1, forward CGTGCAGAGAGAATTCTGAGTTC and reverse AGACGCTTTACGTAGTGCTC and β-actin, forward CCTGTATGCCTCTGGTCGTA and reverse CCATCTCTTGCTCGAAGTCT. Relative amount of RNA was determined using the comparative 2^−∆∆CT^ method and normalized to the level of β-actin expression, as we previously described [38].

### 2.8. HO Activity

Cells were collected, sonicated in phosphate buffer (100 mM; pH 7.4) containing MgCl_2_ (2 mM), and centrifuged at 18,000 g at 4 °C for 15 min. Supernatants were harvested and an aliquot added to a reaction mixture consisting of glucose-6-phosphate (2 mM), glucose-6-phosphate dehydrogenase (0.2 U), NADPH (0.8 mM), rat liver cytosol (2 mg), and the substrate hemin (20 µM). The reaction was conducted in the dark for one hour at 37 °C and extracted bilirubin calculated by the difference in optical density between 464 and 530 nm using an extinction coefficient of 40 mM^−1^cm^−1^ [38].

### 2.9. HO-1 Promoter Activity

Promoter activity was measured using HO-1 promoter-driven luciferase reporters that consisted of a wild-type enhancer (E1) containing three antioxidant responsive elements (AREs) coupled to a minimum HO-1 promoter and a mutant enhancer (M739) that possesses mutations in its three ARE sequences. In brief, cells were transfected with the wild-type or mutant enhancer using lipofectamine for 48 h followed by incubation of cells with an antiviral agent for 8 h. Promoter activity was evaluated by measurement of firefly luciferase activity relative to the internal control TK-renilla luciferase activity using the Dual Luciferase Assay system and a Glomax luminometer (Promega, Madison, WI, USA) [39].

### 2.10. Chromatin Immunoprecipitation (ChIP) Assay

ChIP assays were carried out using a commercially available kit (UpState Biotechnology, Lake Placid, NY, USA) as previously described [40], with some minor modifications. Briefly, cells were lysed and chromatin cross-linked with formaldehyde (1%). Cell lysates were sonicated at 4 °C, centrifuged, and soluble chromatin precleared by incubation with sheared salmon sperm DNA-protein agarose A. Samples were then centrifuged, and a portion of the precleared chromatin was stored and used as input DNA and the remainder incubated with a Nrf2 antibody overnight at 4 °C. Immunoprecipitants were washed, and protein-DNA complexes eluted from the antibody with elution buffer and formaldehyde cross-links reversed by addition of NaCl (5 mM) and heating at 65 °C for 4 h. DNA was extracted and used as a template for qPCR using a primer pair that spanned the HO-1 E1 enhancer: forward, 5′-AAGAGCTCCACCCCCACCCA-3′ and reverse 5′-GGGCTAGCATGCGAAGTGAG-3′. Immunoprecipitated DNA was normalized with respect to its respective chromatin input.

### 2.11. Intracellular Reactive Oxygen Species (ROS) Measurement

Intracellular ROS levels were determined by fluorimetry using the redox-sensitive probe CM-H_2_DCFDA [30,41]. SMCs were washed three times with PBS and then stained with the dye (10 µM) for 30 min at 37 °C in the dark. ROS was quantified by microplate fluorimetry with excitation at 490 nm and emission and 530 nm. Mean values from each treatment were expressed relative to untreated, control cells.

### 2.12. Small Interfering RNA (siRNA) Transfection

Gene expression was silenced using siRNA targeting HO-1 obtained from Dharmacon (Lafayette, CO, USA). Cells were transfected with siRNA specific for HO-1 or non-targeting scrambled (NT) siRNA (100 nM) using lipofectamine [30].

### 2.13. Statistical Analysis

The data are presented as means ± SEM of biological replicates. Statistical analyses were performed using an analysis of variance with the Holm–Sidak post hoc test when more than two treatment groups were compared. A non-parametric Kruskal–Wallis test, followed by a Dunn’s multiple comparison test was used when comparing multiple independent groups and normality assumptions were not met. A value of *p* less than 0.05 was considered significant.

## 3. Results

**Figure 1:**
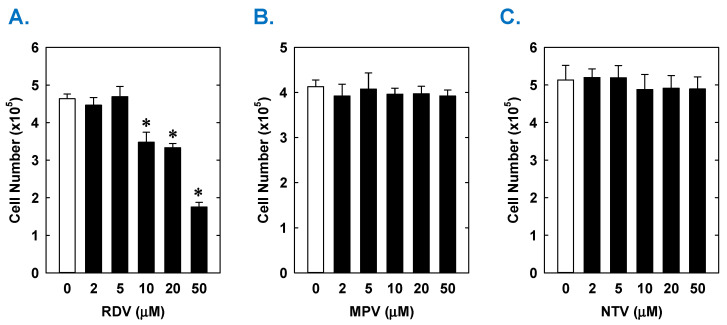
Effect of COVID-19 antiviral drugs on the proliferation of rat aortic SMCs. (**A**) RDV inhibits the proliferation of SMCs in a concentration-dependent manner. (**B**) MPV has no effect on the proliferation of SMCs. (**C**) NTV has no effect on the proliferation of SMCs. Cells were treated with RDV, MPV, or NTV (0–50 µM) for three days. Results are means ± SEM (n = 6). Statistical analysis was performed using an analysis of variance with the Holm–Sidak post hoc test. * Statistically significant effect of RDV.

**Figure 2:**
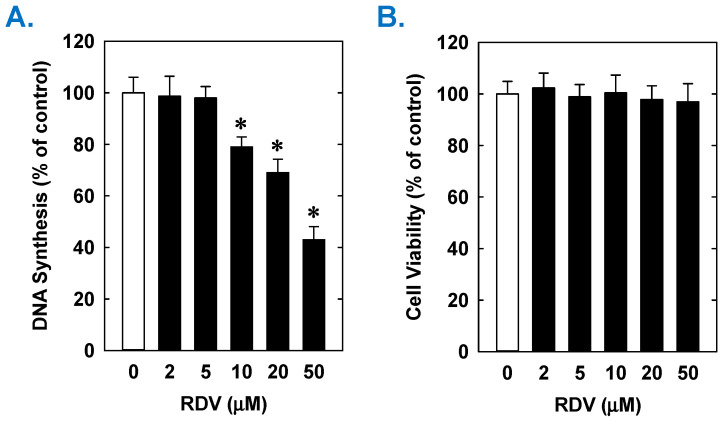
Effect of RDV on DNA synthesis and viability of rat aortic SMCs. (**A**) RDV inhibits DNA synthesis in a concentration-dependent manner. (**B**) RDV does not alter cell viability as assessed by lactate dehydrogenase activity measurements. Cells were incubated in the absence or presence of RDV (0–50 µM) for one day. Results are means ± SEM (n = 6). Statistical analysis was performed using an analysis of variance with the Holm–Sidak post hoc test. * Statistically significant effect of RDV.

**Figure 3:**
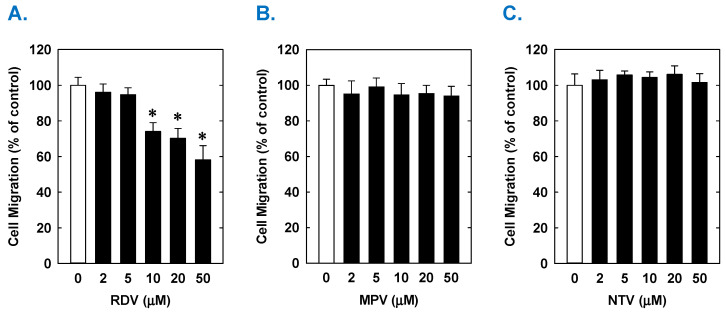
Effect of COVID-19 antiviral drugs on the migration of rat aortic SMCs. (**A**) RDV inhibits the migration of SMCs in a concentration-dependent manner. (**B**) MPV has no effect on the migration of SMCs. (**C**) NTV has no effect on the migration of SMCs. Cells were treated with RDV, MPV, and NTV (0–50 µM) for one day. Results are means ± SEM (n = 6). Statistical analysis was performed using an analysis of variance with the Holm–Sidak post hoc test. * Statistically significant effect of RDV.

**Figure 4:**
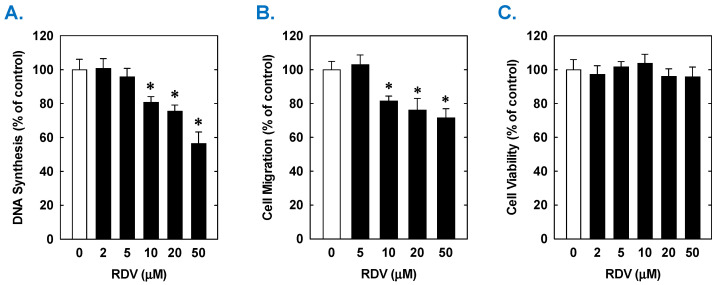
Effect of RDV on the proliferation, migration, and viability of human aortic SMCs. (**A**) RDV inhibits the proliferation of SMCs in a concentration-dependent manner. (**B**) RDV inhibits the migration of SMC in a concentration-dependent manner. (**C**) RDV does not alter cell viability. Cells were treated with RDV, MPV, or NTV (0–50 µM) for one (migration and viability) or three (proliferation) days. Results are means ± SEM (n = 6). Statistical analysis was performed using an analysis of variance with the Holm–Sidak post hoc test. * Statistically significant effect of RDV.

Treatment of rat aortic SMCs with RDV caused a concentration-dependent decrease in cell proliferation (Figure 1A). A significant reduction in cell growth was noted at 10 µM, a concentration that is obtained in the plasma of patients treated with RDV [42]. In contrast, pharmacologically relevant concentrations of MPV (~10 µM) or NTV (~5 µM) had no effect on SMC proliferation [43,44] (Figure 1B,C). Similarly, higher concentrations of MPV or NTV failed to block SMC growth. The antiproliferative effect of RDV was associated with a significant decrease in SMC DNA synthesis, but it had no effect on cell viability (Figure 2A,B). RDV also inhibited the migration of rat aortic SMCs (Figure 3A), whereas MPV and NTV had no effect on SMC motility (Figure 3B,C). Pharmacologically relevant concentrations of RDV also blocked the proliferation and migration of human aortic SMCs, without influencing cell survival (Figure 4).

**Figure 5:**
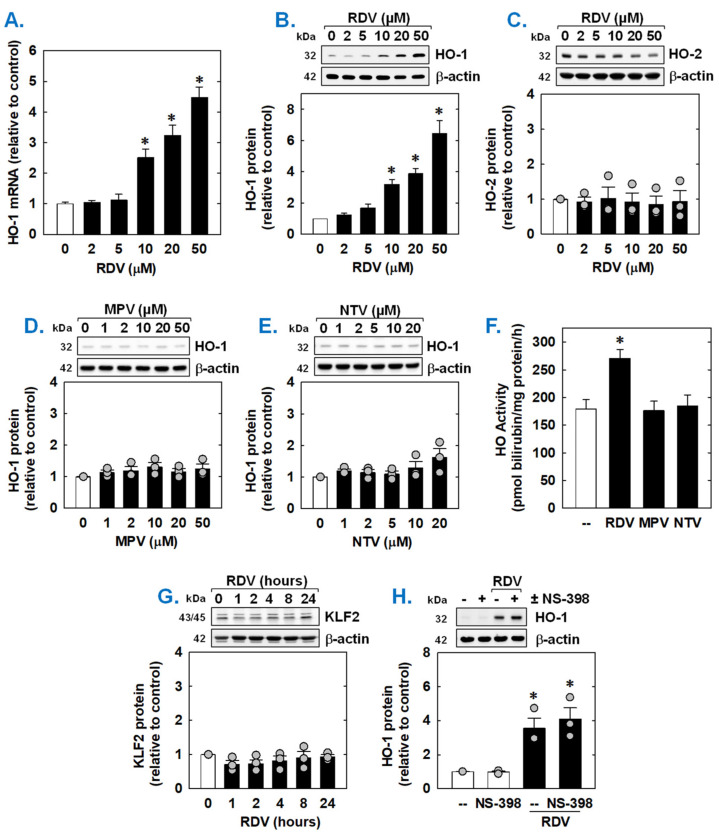
Effect of COVID-19 antiviral drugs on HO-1 expression in rat aortic SMCs. (**A**) RDV exposure for 8 h stimulates a concentration-dependent increase in HO-1 mRNA in SMCs. (**B**) RDV exposure for 24 h stimulates a concentration-dependent increase in HO-1 protein in SMCs. (**C**) RDV exposure for 24 h fails to stimulate HO-2 protein in SMCs. (**D**) MPV exposure for 24 h fails to stimulate HO-1 protein in SMCs. E. NTV exposure for 24 h fails to stimulate HO-1 protein in SMCs. (**E**) RDV (50 µM), but not MPR (50 µM) or NTV (50 µM), exposure for 24 h increases HO activity in SMCs. (**F**) RDV (50 µM), but not MPR (50 µM) or NTV (50 µM), exposure for 24 h increases HO activity in SMCs. (**G**). RDV (50 µM) exposure (0–24 h) does not stimulate the expression of KLF2 in SMCs. (**H**) The selective COX-2 inhibitor NS-398 (10 µM) has no effect on the induction of HO-1 by RDV (50 µM for 24 h). NAC or NS-398 was administered one hour prior to the administration of RDV and was present during the incubation with RDV. Results are means ± SEM (n = 3–6). Statistical analysis was performed using an analysis of variance with the Holm–Sidak post hoc test (**A**,**C**–**H**) or the Kruskal–Wallis test, followed by Dunn’s multiple comparison test (**B**). * Statistically significant effect of RDV.

Since HO-1 is a known modulator of SMC function [45,46,47], the ability of COVID-19 directed antiviral drugs to regulate the expression of HO-1 was examined. Incubation of rat aortic SMCs with RDV stimulated a concentration-dependent increase in HO-1 mRNA and protein (Figure 5A,B). Notably, the induction of HO-1 expression was detected at a clinically relevant concentration (10 µM). In contrast, RDV failed to stimulate the expression of HO-2 protein (Figure 5C). Neither MPV nor NTV induced the expression of HO-1 protein (Figure 5D,E). In addition, RDV evoked a marked increase in HO activity, while MPV and NTV had no effect on HO activity (Figure 5F). Since overexpression of the transcription factor Kruppel-like factor 2 (KLF2) promotes the activation of Nrf2 and the expression of Nrf2-dependent genes [48], we examined whether RDV stimulates HO-1 expression by increasing the expression of KLF2. However, RDV failed to elevate KLF2 expression (Figure 5G). In addition, as the cyclooxygenase-2 (COX-2)-dependent generation of prostacyclin and prostaglandin E2 has been shown to induce the expression of HO-1 in vascular cells [49,50], we determined whether this pathway contributes to HO-1 expression by RDV. Incubation of SMCs with the selective COX-2 inhibitor NS-398 [51] for one hour prior to and during RDV-exposure did not affect the induction of HO-1 by RDV, suggesting that COX-2 is not involved in this process (Figure 5H).

**Figure 6:**
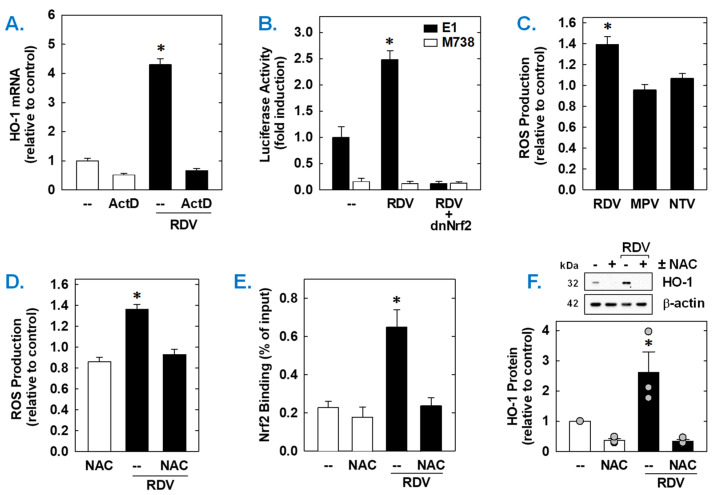
RDV stimulates HO-1 promoter activity via the Nrf2/ARE complex in rat aortic SMCs. (**A**) RDV-mediated HO-1 mRNA expression is dependent on de novo RNA synthesis. SMCs were treated with actinomycin D (ActD; 1 µg/mL) for 8 h in the absence or presence of RDV (50 µM). (**B**) RDV stimulates HO-1 promoter activity. Cells were transfected with a HO-1 promoter construct (E1) or a mutated HO-1 construct (M739) and a Renilla luciferase construct, treated with RDV (50 µM) for 8 h, and then analyzed for luciferase activity. In some instances, a dominant-negative Nrf2 (dnNrf2) construct was co-transfected into cells. (**C**) RDV (50 µM), but not MPR (50 µM) or NTV (50 µM), exposure for 24 h increases ROS production in SMCs. (**D**) RDV (50 µM) exposure for 24 h stimulates ROS production that is blocked by the antioxidant N-acetyl-L-cysteine (NAC; 10 mM). (**E**) ChIP assays demonstrate that RDV (50 µM) exposure for 8 h increases Nrf2 binding to the HO-1 enhancer E1 that is blocked by NAC (10 mM). (**F**) NAC (10 mM) inhibits RDV (50 µM for 24 h)-mediated HO-1 protein expression. Statistical analysis was performed using an analysis of variance with the Holm–Sidak post hoc test. * Statistically significant effect of RDV.

Both the basal expression and induction of HO-1 mRNA by RDV was dependent on de novo RNA synthesis as this response was blocked by the transcriptional inhibitor actinomycin D (Figure 6A). RDV also activated the HO-1 promoter; however, mutation of the ARE sequences abrogated the activation of the promoter (Figure 6B). Elimination of the ARE sequences also strikingly attenuated baseline promoter activity. As Nrf2 contributes to ARE-driven expression of antioxidant enzymes, including HO-1 [38,39,40], the involvement of this transcription factor was examined. Transfection of SMCs with a dominant-negative mutant of Nrf2 that had its activation domain deleted inhibited basal HO-1 promoter activity and the RDV-mediated rise in promoter activity (Figure 6B).

Subsequently, the upstream signaling cascade involved in the induction of HO-1 by RDV was investigated. Since oxidative stress is an established inducer of HO-1 [22,23,24], the role of ROS in the induction of HO-1 was determined. Treatment of vascular SMCs with RDV elicited a significant increase in intracellular ROS that was not seen with MPV or NTV (Figure 6C). Exposure of cells with the antioxidant N-acetyl-L-cysteine (Figure 6C) for one hour prior and during RDV-treatment prevented the rise in ROS generation (Figure 6D). ChIP assays further revealed that Nrf2 binding to the HO-1 enhancer E1 was substantially enriched after treatment with RDV (Figure 6E). However, N-acetyl-L-cysteine pretreatment also blocked the RDV-mediated binding of Nrf2 to the E1 enhancer of the HO-1 promoter (Figure 6D) and the induction of HO-1 by RDV (Figure 6F).

**Figure 7:**
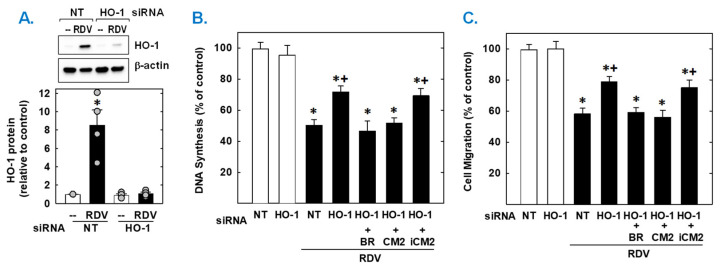
HO-1 contributes to the antiproliferative and antimigratory effect of RDV. (**A**) HO-1 expression in vascular SMCs transfected with HO-1 (100 nm) or non-targeting (NT; 100 nm) siRNA and then treated with RDV (50 µM) for 24 h. (**B**,**C**) HO-1 knockdown decreases RDV-mediated inhibition of SMC DNA synthesis and migration. Cells were transfected with HO-1 (100 nm) or NT (100 nm) siRNA and then treated with RDV (50 µM) in the absence or presence of bilirubin (20 µM), CORM2 (CM2; 20 µM), or iCORM2 (iCM2; 20 µM). Results are means ± SEM (4–6). Statistical analysis was performed using an analysis of variance with the Holm–Sidak post hoc test (**B**,**C**) or the Kruskal–Wallis test, followed by Dunn’s multiple comparison test (**A**). * Statistically significant effect of RDV. ^+^ Statistically significant effect of HO-1 siRNA.

Finally, the functional importance of the induction of HO-1 by RDV was established by knocking down HO-1 expression. Transfection of vascular SMCs with HO-1 siRNA inhibited the RDV-mediated increase in HO-1 expression and partially rescued the proliferation and migration of RDV-treated SMCs (Figure 7). In contrast, NT siRNA was unable to prevent the induction of HO-1 in response to the antiviral agent. In the absence of RDV, HO-1 or NT siRNA had no effect on SMC function. Next, we determined which of the HO-1 products could restore the SMC response in HO-1 silenced cells. Exogenous administration of either the CO donor (CORM2) or bilirubin could substitute for the deficit in HO-1 and fully restore the antiproliferative and antimigratory action of RDV. However, the inactive CO donor (iCORM2) was incapable of restoring the actions of the drug.

## 4. Discussion

In the current study, we identified RDV as a novel inhibitor of vascular SMC proliferation and migration. These effects seem to be exclusive to remdesivir as other SARS-CoV-2-directed antivirals, including MPV and NTV, had no effect on SMC function. The antiproliferative and antimigratory actions of RDV are seen in vascular SMCs isolated from rat and human arteries. In addition, we found that RDV stimulates HO-1 gene expression in vascular SMCs, while MPV and NTV fails to increase HO-1 expression or activity. The induction of HO-1 by RDV requires the production of ROS and involves the activation of the Nrf2-ARE signaling complex. Importantly, the induction of HO-1 participates in the antiproliferative and antimigratory action of RDV by generating CO and/or bilirubin. The ability of RDV to evoke these pleiotropic effects on SMCs may mitigate the development occlusive vascular disease in patients exposed to SARS-CoV-2 and suggest an extra potential value of RDV compared to other antivirals targeting this virus.

This is the first study to show that RDV blocks vascular SMC proliferation and migration. These actions of RDV are concentration-dependent and, pointedly, are observed at concentrations found in the plasma of individuals treated with this antiviral compound [42]. Healthy adult volunteers receiving RDV attained peak concentrations of approximately 10 µM. However, even higher plasma concentrations of RDV can be achieved in patients with renal disease and/or with concurrent application of inhibitors of esterases and cytochrome P450 enzymes that reduce drug clearance. Thus, our results demonstrate a clear potential for RDV to alter SMC function. The antiproliferative and antimigratory effect of RDV is observed in both human and rodent vascular SMCs, illustrating that the drug slows SMC growth and motility across animal species. Moreover, these actions of RDV occur in the absence of cell death and are accompanied by a pronounced decline in DNA synthesis, indicating that RDV functions in a cytostatic rather cytotoxic manner. Our finding that RDV inhibits SMC replication is in-line with earlier studies demonstrating that pharmacologically relevant concentrations of RDV blocks proliferation and DNA synthesis in cardiac and kidney cells as well as fibroblasts [52,53]. In addition, RDV inhibits the migration of primary mouse and human fibroblasts [44]. As we observed in SMCs, the antiproliferative and antimigratory effect of RDV in these cells also occurs in the absence of cell death. However, clinical attainable concentrations of RDV have been shown to decrease the viability of a human hepatoma ovarian cancer cell line, while preclinical testing studies found that chronic exposure of RDV for up to 14 days evokes concentration-dependent toxic responses in various primary human cell types and immortalized human cell lines [54,55,56]. Thus, the cytotoxic effect of RDV appears to be cell-specific and concentration- and time-dependent. The ability of RDV to suppress vascular SMC proliferation and migration may limit lesion formation in the vasculature and abate the progression of vascular occlusive disease in patients with COVID-19.

The study also identified the heme-metabolizing enzyme HO-1 as an off-target of RDV. Incubation of vascular SMCs with RDV triggers an increase in HO-1 expression and activity that is not observed with either MPV or NTV. Moreover, RDV does not stimulate the expression of HO-2, demonstrating that RDV selectivity induces the expression of the HO-1 isoform. Notably, the ability of RDV to elevate HO-1 levels is observed at concentrations (10 µM) found in patients receiving RDV [42]. Interestingly, biomechanical forces generated by interstitial fluid flow can sensitize cells to inducers of HO-1, affording a possible process wherein the potency of RDV is elevated in vivo [57]. The induction of HO-1 by RDV requires de novo RNA synthesis and likely arises from the transcriptional activation of the gene since the drug directly activates the HO-1 promoter. However, it does not involve the generation of prostaglandins by COX-2 as the selective COX-2 inhibitor NS-398 [51] fails to mitigate RDV-mediated HO-1 expression. The activation of the HO-1 promoter involves the AREs since mutation of this element abrogates reporter activation in response to RDV. Interestingly, RDV increases Nrf2 binding to the ARE as measured by the ChIP assay. The activation of Nrf2 by RDV noted in our study is consistent with an earlier report showing that RDV stimulates an increase in the expression and nuclear translocation of Nrf2 in a line of immortalized kidney epithelial cells [56]. In addition, we found that transfection of SMCs with a dominant-negative mutant of Nrf2 blocks the activation of the HO-1 promoter by RDV. Together, these findings demonstrate an essential role for Nrf2 in mediating RDV-induced HO-1 gene transcription in vascular SMCs. Moreover, the ability of RDV to activate Nrf2 in other cell types, suggests the RDV may induce the expression of HO-1 beyond the vasculature.

The capacity of RDV to induce HO-1 gene expression is dependent on the cellular redox potential. Treatment of vascular SMCs with RDV evokes a significant rise in ROS production and blockade of this oxidative response by the antioxidant N-acetyl-L-cysteine prevents the activation of Nrf2 and the induction of HO-1 by RDV. The capacity of RDV to elicit ROS production has also been verified in cardiomyocytes, renal proximal tubular epithelial cells, and in hepatic and ovarian cancer cell lines [52,55,56]. As observed in cardiac and hepatic cells, RDV evoked a rise in ROS following a 24 h incubation period while a longer 72 h exposure interval induced ROS formation in a human ovarian cancer cell line [52,55,56]. In addition, the in vivo administration of RDV produces oxidative stress in rodents [58]. The inability of MPV or NTV to stimulate ROS synthesis in SMCs likely underlies their failure to increase HO-1 expression. Little is known regarding the ability of these two antiviral drugs to generate ROS in other cells. Extremely high concentrations of NTV approaching the mM range causes oxidative stress in the crustacean Ceriodaphnia dubia while much lower concentrations of NTV (10–60 µM) in combination with ritonavir provoke ROS formation in chondrocytes [58,59]. However, it is unclear whether NTV is responsible for ROS production in the chondrocyte study since ritonavir is a known producer of superoxide and may account for the increase in ROS formation in these cells [60]. Molecular analysis of electron acceptor and donor capacity suggest that MPV possesses antioxidant properties, but additional studies are needed to assess the ability of this drug to affect redox balance [61,62].

Although the intracellular source of RDV-mediated ROS formation remains to be fully demarcated, a mitochondrial origin is likely. RDV, as with many other nucleoside analogs, has been demonstrated to disrupt mitochondrial function. RDV affects mitochondrial ROS formation, respiration, energetics, dynamics, redox potential, membrane potential, DNA and protein content, and interacts with mammalian RNA and DNA polymerase [52,53,54,55,63,64]. Crucially, RDV inhibits the activity of mitochondrial complex I and III which lead to the generation of superoxide [65]. Moreover, the potent inhibitor of mitochondrial complex III antimycin A mimics many of the mitochondrial defects provoked by RDV, highlighting the importance of this multi-subunit transmembrane protein in mediating the biological actions of the antiviral drug [52]. The mechanism by which RDV activates Nrf2 is not exactly known. It does not involve an increase in KLF2 expression or COX-2 activity and likely entails the oxidation of cysteine(s) in Kelch-like erythroid cell-derived protein-1 (Keap1) and the subsequent dissociation of Nrf2 from Keap-1 and/or blockade of Keap1-mediated ubiquitination and destruction of Nrf2 [66].

Meaningfully, the upregulation of HO-1 impacts the antiproliferative and antimigratory effects of RDV. Knocking down HO-1 expression increases the proliferation and migration of vascular SMCs exposed to RDV. These findings are consistent with former studies indicating that HO-1 represses the growth and movement of SMCs [49,50,51]. In addition, exogenous administration of CO and bilirubin substitutes for the loss of HO-1 and restores the antiproliferative and antimigratory response of RDV-treated SMCs. These studies agree with previous work from our laboratory and others showing that both heme metabolites negatively influence SMC function [37,47,67,68]. However, knocking down HO-1 expression does not fully regain cell function, indicating that other factors also mediate the antiproliferative and antimigratory effects of RDV. In this respect, activation of Nrf2 may result in the expression of other antioxidant enzymes that attenuate SMC function [57,69,70]. Furthermore, RDV blocks mitochondrial complex I activity which has been implicated in promoting SMC proliferation and migration both in vitro and in vivo [65,71]. Moreover, RDV may obviate SMC function by suppressing mitochondrial energy production, DNA-polymerase activity, and/or transforming growth factor-β signaling [52,53]. Thus, RDV may interfere with SMC proliferation and migration through multiple discrete mechanisms.

The activation of the Nrf2-HO-1 signaling axis by RDV may evoke several important actions that further contributes to the salutary pharmacological profile of the drug in COVID-19 patients. The Nrf2-HO-1 axis exerts beneficial effects during virus infection by limiting oxidative and inflammatory stress and promoting the survival of host cells via the generation of CO and bilirubin [72]. Intriguingly, the Nrf2-HO-1 signaling pathway is suppressed in biopsies acquired from COVID-19 patients or in multiple cell lines infected with SARS-CoV-2 [73,74]. Moreover, studies performed in mouse models of SARS-CoV-2 infection found that ablation of Nrf2 is associated with exacerbated clinical disease as reflected by a greater loss of body weight, increases in lung inflammation, and higher peak SARS-CoV-2 titers in the lung [75]. Hence, the ability of RDV to increase Nrf2 signaling may enhance important immune and anti-inflammatory responses in COVID-19. Furthermore, a role for HO-1 in mediating the actions of Nrf2 is supported by previous work showing that HO-1 blocks inflammation and promotes antiviral immunity against a wide range of viruses [75,76]. The induction of HO-1 by RDV may also ameliorate the vasculopathy that develops during COVID-19. Aside from limiting arterial lesion formation via the blockade of vascular SMC proliferation and migration, HO-1 also prevents endothelial inflammation and injury that underlies the multiorgan complications of COVID-19 [26]. In addition, HO-1 exerts potent antithrombotic effects that may suppress the elevated risk for deep vein thrombosis, pulmonary embolism, and stroke in patients with COVID-19 [3,77,78].

While this in vitro cell-based study provides important new information regarding the cellular and molecular actions of RDV in vascular SMCs, it does not fully explore the effects of RDV on SMC function, nor does it mimic the complex biochemical and biophysical interactions that transpire in vivo. Since alterations in vascular contractile function have been reported in COVID-19, it will be valuable to extend this work and assess the actions of RDV on vascular reactivity using various ex vivo models [17]. In addition, potential effects of RDV on endothelial cell function should be investigated given the reported malfunction of these cells in COVID-19 patients [8,9,10]. Moreover, this work should be expanded to examine for possible beneficial effects of RDV in both vascular and immune cells exposed to inflammatory stress, a key mediator of pathology in COVID-19 [2,3,4]. It will also be critical to explore if RDV improves the aberrant vascular remodeling response seen in animal models of arterial injury or disease. The utility of RDV in ameliorating vascular dysfunction in animal models of COVID-19 should also be pursued [79]. Finally, studies utilizing HO-1-deficient mice will be crucial in authenticating a role for this enzyme in contributing to the cardiovascular actions of RDV [80].

In conclusion, the current study discovered that RDV is an inhibitor of vascular SMC proliferation and migration, and that RDV stimulates the expression of HO-1 via the ROS-Nrf2 signaling cascade. Interestingly, these effects are exclusive to RDV and are not noted with other SARS-CoV-2-directed antiviral drugs. In addition, it found that HO-1 supports the antimigratory and antiproliferative actions of RDV by synthesizing CO and bilirubin. The capability of RDV to initiate these multifaceted effects on vascular SMCs may uniquely limit the development of vascular disease in patients with COVID-19 when compared to other antiviral agents. The study also highlights the therapeutic potential of targeting the Nrf2-HO-1 signaling pathway in COVID-19.

## Data Availability

The original contributions presented in this study are included in the article. Further inquiries can be directed to the corresponding author.

## References

[1] World Health Organization (2023). WHO Coronavirus Disease (COVID-19) Dashboard. https://data.who.int/dashboards/covid19/deaths?n=0.

[2] Tay M.Z., Poh C.M., Renia L., MacAry P.A., Ng L.F.P. (2020). The trinity of COVID-19: Immunology, inflammation and intervention. Nat. Rev. Immunol..

[3] McFadyen J.D., Stevens H., Peter K. (2020). The emerging threat of (micro) thrombosis in COVID-19 and its therapeutic implications. Circ. Res..

[4] Nazerian Y., Ghasemi M., Yassaghi Y., Nazerian A., Hashemi S.M. (2022). Role of SARS-CoV-2-induced cytokine storm in multi-organ failure: Molecular pathways and potential therapeutic options. Int. Immunopharmacol..

[5] Morrow A.J., Sykes R., McIntosh A., Kamdar A., Bagot C., Bayes H.K., Blyth K.G., Briscoe M., Bulluck H., Carrick D. (2022). A multisystem, cardio-renal investigation of post-COVID-19 illness. Nat. Med..

[6] Onder G., Rezza G., Brusaferro S. (2020). Case-fatality rate and characteristics of patients dying in relation to COVID-19. JAMA.

[7] Guo Y.-R., Cao D.-Q., Hong Z.-S., Tan Y.-Y., Chen S.-D., Jin H.-J., Tan K.S., Wang D.-Y., Yan Y. (2020). The origin, transmission, and clinical therapies on coronavirus disease 2019 (COVID-19) outbreak—An update on the status. Mil. Med. Res..

[8] Libby P., Luscher T. (2020). COVID-19 is, in the end, an endothelial disease. Eur. Heart J..

[9] Ma Z., Yang K.Y., Huang Y., Liu K.O. (2022). Endothelial contribution to COVID-19: An update on mechanisms and therapeutic implications. J. Mol. Cell. Cardiol..

[10] Prasad M., Leon M., Lerman L.O., Lerman A. (2021). Viral endothelial dysfunction: A unifying mechanism for COVID-19. Mayo Clin. Proc..

[11] McCraken I.R., Saginc G., He L., Huseynov A., Daniels A., Fletcher S., Peghaire C., Kalna V., Andaloussi-Mae M., Muhl L. (2021). Lack of evidence of angiotensin-converting enzyme 2 expression and replicative infection by SARS-CoV-2 in human endothelial cells. Circulation.

[12] Marchiano S., Hsiang T.Y., Khanna A., Higashi T., Whitmore L.S., Bargehr J., Davaapil H., Chang J., Smith E., Ong L.P. (2021). SARS-CoV-2 infects human pluripotent stem cell-derived cardiomyocytes, impairing electrical and mechanical function. Stem. Cell Rep..

[13] Suzuki Y.J., Nikolaienko S.I., Dibrova V.A., Dibrova Y.V., Vasylyk V.M., Novikov M.Y., Shults N.V., Gychka S.G. (2021). SARS-CoV-2 spike protein-mediated cell signaling in lung vascular cells. Vasc. Pharmacol..

[14] Martinez-Salazar B., Holwerda M., Studle C., Piragyte I., Mercader N., Engelhardt B., Rieben R., Doring Y. (2022). COVID-19 and the vasculature: Current aspects and long-term consequences. Front. Cell Devel. Biol..

[15] Tzankov A., Bhattacharyya S., Kotlo K., Tobacman J.K. (2022). Increase in chondroitin sulfate and decline in arylsulfatase B may contribute to pathophysiology of COVID-19 respiratory failure. Pathobiology.

[16] Zanoli L., Gaudio A., Mikhailidis D.P., Katsiki N., Castellino N., Cicer L.L., Geraci G., Sessa C., Fiorito L., Marino F. (2022). Vascular dysfunction of COVID-19 is partially reversed long-term. Circ. Res..

[17] Sykes R.A., Neves K.B., Alves-Lopez R., Caputo I., Fallon K., Jamieson N.B., Kamdar A., Legrini A., Leslie H., McIntosh A. (2023). Vascular mechanisms of post-COVID-19 conditions: Rho-kinase is a novel target for therapy. Eur. Heart J..

[18] Islam T., Hasan M., Rhamna M.S., Islam M.R. (2022). Comparative evaluation of authorized drugs for treating COVID-19 patients. Health Sci. Rep..

[19] Yin W., Mao C., Luan X., Shen D.D., Shen Q., Su H., Wang X., Zhou F., Zhao W., Gao M. (2020). Structural basis for the inhibition of RNA-dependent RNA polymerase from SARS-CoV-2 by remdesivir. Science.

[20] Gordon C.J., Tchesnokov E.P., Schinazi R.F., Gotte M. (2021). Molnupiravir promotes SARS-CoV-2 mutagenesis vi the RNA template. J. Biol. Chem..

[21] Owen D.R., Allerton C.M.N., Anderson A.S., Aschenbrenner L., Avery M., Berritt S., Boras B., Cardin R.D., Carlo A., Coffman K.J. (2021). An oral SARS-CoV-2 M(pro) inhibitor candidate for the treatment of COVID-19. Science.

[22] Ayer A., Zarjou A., Agarwal A., Stocker R. (2016). Heme oxygenases in cardiovascular health and disease. Physiol. Rev..

[23] Durante W., Johnson F.K., Johnson R.A. (2006). Role of carbon monoxide in cardiovascular function. J. Cell. Mol. Med..

[24] Durante W. (2020). Targeting heme oxygenase-1 in the arterial response to injury and disease. Antioxidants.

[25] Durante W. (2011). Protective role of heme oxygenase-1 against inflammation in atherosclerosis. Front. Biosci..

[26] Espinosa J.A., Gonzalez P.A., Kalergis A.M. (2017). Modulation of antiviral immunity by heme oxygenase-1. Am. J. Pathol..

[27] Wagener F.A.D.T.G., Pickkers P., Peterson S.J., Immenschuh S., Abraham N.G. (2020). Targeting the heme-heme oxygenase system to prevent severe complications following COVID-19 infections. Antioxidants.

[28] Singh D., Wasan H., Reeta K.H. (2020). Heme oxygenase-1 modulation: A potential therapeutic target for COVID-19 and associated complications. Free Radic. Biol. Med..

[29] Rossi M., Piagnerelli M., Van Meerhaeghe A., Boudjeltia K.Z. (2020). Heme oxygenase-1 (HO-1) cytoprotective pathway: A potential treatment strategy against coronavirus disease 2019 (COVID-19)-induced cytokine storm. Med. Hypotheses.

[30] Liu X.M., Durante Z.E., Peyton K.J., Durante W. (2016). Heme oxygenase-1-derived bilirubin counteracts HIV protease inhibitor-mediated endothelial dysfunction. Free Radic. Biol. Med..

[31] Ben-Romano R., Rudich A., Etzion S., Potashnik R., Kagan E., Greenbaum U., Bashan N. (2002). Nelfinavir induces adipocyte insulin resistance through induction of oxidative stress: Differential protective effect of antioxidant agents. Antiviral Ther..

[32] Muhl H., Paulukat J., Hofler S., Hellmuth M., Franzen R., Pfeilschifter J. (2004). The HIV protease inhibitor ritonavir synergizes with butyrate for the induction of apoptotic cell death and mediates expression of heme oxygenase-1 in DLD-1 colon carcinoma cells. Br. J. Pharmacol..

[33] Laurence J., Elhadad S., Gostynska S., Yu Z., Terry H., Varshney R., Fung K.-M., Choi M.E., Ahamed J. (2020). HIV protease inhibitor ritonavir induces renal fibrosis and dysfunction: Role of platelet-derived TGF-β1 and intervention via antioxidant pathway. AIDS.

[34] Durante W., Schini V.B., Catovsky S., Kroll M.H., Vanhoutte P.M., Schafer A.I. (1993). Plasmin potentiates induction of nitric oxide synthesis by interleukin-1 beta in vascular smooth muscle cells. Am. J. Physiol..

[35] Peyton K.J., Reyna S.V., Chapman G.B., Ensenat D., Liu X.M., Wang H., Schafer A.I., Durante W. (2002). Heme oxygenase-derived carbon monoxide is an autocrine inhibitor of vascular smooth muscle cell growth. Blood.

[36] Peyton K.J., Yu Y., Yates B., Shebib A.R., Liu X.M., Wang H., Durante W. (2011). Compound C inhibits vascular smooth muscle cell proliferation and migration in an AMPK-activated protein kinase-independent fashion. J. Pharmacol. Exp. Ther..

[37] Peyton K.J., Shebib A.R., Azam A.M., Liu X.M., Tulis D.A., Durante W. (2012). Bilirubin inhibits neointima formation and vascular smooth muscle cell proliferation and migration. Front. Pharmacol..

[38] Liu X.M., Peyton K.J., Durante W. (2013). Physiologic cyclic strain promotes endothelial cell survival via the induction of heme oxygenase-1. Am. J. Physiol. Heart Circ. Physiol..

[39] Liu X.M., Peyton K.J., Ensenat D.E., Wang H., Schafer A.I., Alam J., Durante W. (2005). Endoplasmic reticulum stress stimulates heme oxygenase-1 gene expression in vascular smooth muscle cells. Role in cell survival. J. Biol. Chem..

[40] Liu X.M., Peyton K.J., Ensenat D., Wang H., Hannink M., Alam J., Durante W. (2007). Nitric oxide stimulates heme oxygenase-1 gene transcription via the Nrf2/ARE complex to promote vascular smooth muscle cell survival. Cardiovasc. Res..

[41] Durante W., Peyton K.J., Schafer A.I. (1999). Platelet-derived growth factor stimulates heme oxygenase-1 gene expression and carbon monoxide production in vascular smooth muscle cells. Arterioscler. Thromb. Vasc. Biol..

[42] Wang Y., Zhang D., Du G., Du R., Zhao J., Jin J., Fu S., Gao L., Cheng Z., Lu Q. (2020). Remdesivir in adults with severe COVID-19: A randomized, double-blind, placebo-controlled multicenter trial. Lancet.

[43] Painter W.P., Holman W., Bush J.A., Almazedi F., Malik H., Eraut N.C.J.E., Morin M.J., Szewczyk L.J., Painter G.R. (2021). Human safety, tolerability, and pharmacokinetics of molnupiravir, a novel broad-spectrum oral antiviral agent with activity against SARS-CoV-2. Antimicrob. Agents Chemother..

[44] Hau R.K., Wright S.H., Cherrington N.J. (2022). PF-07321332 (Nirmatrelvir) does not interact with human ENT1 or ENT2: Implications for COVID-19 patients. Clin. Transl. Sci..

[45] Duckers H.J., Boehm M., True A.L., Yet S.F., San H., Park J.L., Clinton Webb R., Lee M.E., Nagel G.J., Nagel E.G. (2001). Heme oxygenase-1 protects against vascular constriction and proliferation. Nat. Med..

[46] Tulis D.A., Durante W., Peyton K.J., Evans A.J., Schafer A.I. (2001). Heme oxygenase-1 attenuates vascular remodeling following balloon injury in rat carotid arteries. Atherosclerosis.

[47] Rodriguez A.I., Gangopadhyay A., Kelly E.E., Pagano P.J., Zuckerbraun B.S., Bauer P.M. (2010). HO-1 and CO decrease platelet-derived growth factor-induced vascular smooth muscle cell migration via inhibition of Nox1. Arterioscler. Thromb. Vasc. Biol..

[48] Fledderus J.O., Boon R.A., Volger O.L., Hurttila H., Yla-Herttuala S., Pannekoek H., Levonen A.-L., Horrevoets A.J.G. (2008). KLF2 primes the antioxidant transcription factor Nrf2 for activation in endothelial cells. Arterioscler. Thromb. Vasc. Biol..

[49] Di Francesco L., Totani L., Dovizio M., Piccoli A., Di Francesco A., Salvatore T., Pandolfi A., Evangelista V., Dercho R.A., Seta F. (2009). Induction of prostacyclin by steady laminar shear stress suppresses tumor necrosis factor-α biosynthesis via heme oxygenase-1 in human endothelial cells. Circ. Res..

[50] Vinals M., Martinez-Gonzalez J., Badimon J.J., Badimon L. (1997). HDL-induced prostacyclin release in smooth muscle cells is dependent on cyclooxygenase-2 (Cox-2). Arterioscler. Thromb. Vasc. Biol..

[51] Futaki N., Takahashi S., Yokoyama M., Arai I., Higuchi S., Otomo S. (1994). NS-398, a new anti-inflammatory agent, selectively inhibits prostacyclin G/H synthase/cyclooxygenase (COX-2) activity in vitro. Prostaglandins.

[52] Merches K., Breunig L., Fender J., Brand T., Bätz V., Idel S., Kollipara L., Reinders Y., Sickmann A., Mally A. (2022). The potential of remdesivir to affect function, metabolism and proliferation of cardiac and kidney cells in vitro. Arch. Toxicol..

[53] Zhang J., Zhang X., Guo X., Li W., Zhang T., Chai D., Liu Y., Chen L., Ai X., Zhou T. (2024). Remdesivir alleviates skin fibrosis by suppressing TGF-β1 signaling pathway. PLoS ONE.

[54] Bjork J.A., Wallace K.B. (2021). Remdesivir; molecular and functional measures of mitochondrial safety. Toxic. Appl. Pharmacol..

[55] Xu Y., Barauskas O., Kim C., Babusis D., Murakami E., Kornyeyev D., Lee G., Stepan G., Perron M., Bannister R. (2021). Off-target in vitro profiling demonstrates that remdesivir is a highly selective antiviral agent. Antimicrob. Agents Chemother..

[56] Lee C.M., Kang M.-A., Bae J.S., Park K., Yang Y.-H., Lee J., Jang K.Y., Park S.-H. (2022). An in vitro study on anti-carcinogenic effect of remdesivir in human ovarian cancer cells via generation of reactive oxygen species. Hum. Exp. Toxicol..

[57] Ali F., Zakkar M., Karu K., Liddington E.A., Hamdulay S.S., Boyle J.J., Zloh M., Bauer A., Haskard D.O., Evans P.C. (2009). Induction of the cytoprotective enzyme heme oxygenase-1 by statins is enhanced in vascular endothelium exposed to laminar shear stress and impaired by disturbed blood flow. J. Biol. Chem..

[58] Abbasi M.M., Darbani R., Rabet O., Ghorbanihaghjo A., Rashtchizadeh N., Raeisi S., Khordadmehr M. (2024). Effects of remdesivir on liver enzymes, oxidative stress and liver histopathology in rats. Int. J. Ecotoxicol. Ecobiol..

[59] Nugnes R., Orlo E., Russo C., Lavorgna M., Isidori M. (2024). Comprehensive eco-geno-toxicity and environmental risk of common antiviral drugs in aquatic environments post pandemic. J. Hazard Mater..

[60] Kong K., Chang Y., Qiao H., Zhao C., Chen X., Rong K., Zhang P., Jin M., Zhang J., Li H. (2022). Paxlovid accelerates cartilage degeneration and senescence through activating endoplasmic reticulum stress and interfering redox homeostasis. J. Transl. Med..

[61] Martinez A. (2021). Electron donor-acceptor capacity of selected pharmaceuticals against COVID-19. Antioxidants.

[62] Yasri S., Wiwanitki V. (2022). Molnupiravir, favipiravir and other antiviral drugs with proposed potentials for management of COVID-19: A concern on antioxidant aspect. Int. J. Biochem. Mol. Biol..

[63] Bartolini D., Stabile A.M., Bastianelli S., Giustarini D., Pierucci S., Busti C., Vacca C., Gidari A., Francisci D., Castronari R. (2021). SARS-CoV2 infection impairs the metabolism and redox function of cellular glutathione. Red. Biol..

[64] Kwok M., Lee C., Li H.S., Deng R., Tsoi C., Ding Q., Tsang S.Y., Leung K.T., Yan B.P., Poon E.N. (2021). Remdesivir induces persistent mitochondrial and structural damage in human induced pluripotent stem cell-derived cardiomyocytes. Cardiovasc. Res..

[65] DeFoor N., Paul S., Li S., Basso E.K.G., Stevenson V., Browning J.L., Prater A.K., Brindley S., Tao G., Pickrell A.A. (2023). Remdesivir increases mtDNA copy number causing mild alterations to oxidative phosphorylation. Sci. Rep..

[66] Fisar Z., Luptak M., Hroudova J. (2021). Little in vitro effect of remdesivir on mitochondrial respiration and monoamine oxidase activity in isolated mitochondria. Tox. Lett..

[67] Zhang D.D., Hannink M. (2003). Distinct cysteine residues in keap1 are required for keap1-dependent ubiquitination of Nrf2 and for stabilization of Nrf2 by chemopreventative agents and oxidative stress. Mol. Cell Biol..

[68] Otterbein L.E., Zuckerbraun B.S., Haga M., Liu F., Song R., Usheva A., Stachulak C., Bodyak N., Smith R.N., Csizmadia E. (2003). Carbon monoxide suppresses artherosclerotic lesions associated with chronic graft rejection and with balloon injury. Nat. Med..

[69] Ollinger R., Bilban M., Erat A., Froio A., McDaid J., Tyagi S., Csizmadia E., Graca-Souza A.V., Liloia A., Soares M.P. (2005). Bilirubin: A natural inhibitor of vascular smooth muscle proliferation. Circulation.

[70] Levonen A.L., Inkala M., Heikura S., Jauhiainen S., Jyrkkanen H.K., Kansanen E., Maatta K., Romppanen E., Turunen P., Rutanen J. (2007). Nrf2 gene transfer induces antioxidant enzymes and suppresses smooth muscle cell growth in vitro and reduces oxidative stress in rabbit aorta in vivo. Arterioscler. Thromb. Vasc. Biol..

[71] Ashino T., Yamamoto M., Yoshida T., Numazawa S. (2013). Redox-sensitive transcription factor Nrf2 regulates vascular smooth muscle cell migration and neointimal hyperplasia. Arterioscler. Thromb. Vasc. Biol..

[72] Yin J., Xia W., Wu M., Zhang Y., Huang S., Zhang A., Jia Z. (2019). Inhibition of mitochondrial complex I activity attenuates neointimal hyperplasia by inhibiting smooth muscle cell proliferation and migration. Chem. Biol. Interact..

[73] Wang Y., Ma J., Jiang Y. (2023). Transcription factor Nrf2 as a potential therapeutic agent for COVID-19. Cell Stress Chap..

[74] Olagnier D., Farahani E., Thyrsted J., Blay-Cadanet J., Herengt A., Idorn M., Hait A., Hernaez B., Knudson A., Iversen M.B. (2020). SARS-CoV2-mediated suppression of Nrf2-signaling reveals potent antiviral and anti-inflammatory activity of 4-octyl-itaconate and dimethyl fumurate. Nat. Commun..

[75] Qu Y., Haas de Mello A., Morris D.R., Jones-Hall Y.L., Ivanciuc T., Sattler R.A., Paessler S., Menachery V.D., Garafolo R.P., Casola A. (2023). SARS-CoV-2 inhibits Nrf2-mediated antioxidant responses in airway epithelial cells and in the lung of a murine model of infection. Microbiol. Spectr..

[76] Ryter S., Choi A.M.K. (2016). Targeting heme oxygenase-1 and carbon monoxide for therapeutic modulation of inflammation. Transl. Res..

[77] Libby P. (2024). Endothelial inflammation in COVID-19: Disrupted endothelial function underlies the multiorgan complications of COVID-19. Science.

[78] True A.L., Olive M., Boehm M., San H., Westrick R.J., Raghavachari N., Xu X., Lynn E.G., Sack M.N., Munson P.J. (2007). Heme oxygenase-1 deficiency accelerates formation of arterial thrombosis through oxidative damage to endothelium, which is rescued by inhaled carbon monoxide. Circ. Res..

[79] Chung J., Pierce J., Franklin C., Olson R.M., Morrison A.R., Amos-Landgraf J. (2025). Translating animal models of SARS-CoV-2 infection to vascular, neurological and gastrointestinal manifestations of COVID-19. Dis. Models Mech..

[80] Poss K.D., Tonegawa S. (1997). Heme oxygenase 1 is required for mammalian iron utilization. Proc. Natl. Acad. Sci. USA.

